# Kernel Fusion Method for Detecting Cancer Subtypes via Selecting Relevant Expression Data

**DOI:** 10.3389/fgene.2020.00979

**Published:** 2020-09-10

**Authors:** Shuhao Li, Limin Jiang, Jijun Tang, Nan Gao, Fei Guo

**Affiliations:** ^1^School of Computer Science and Technology, College of Intelligence and Computing, Tianjin University, Tianjin, China; ^2^Department of Computer Science and Engineering, University of South Carolina, Columbia, SC, United States; ^3^School of Computer Science and Technology, Zhejiang University of Technology, Hangzhou, China

**Keywords:** cancer subtype, similarity Kernel fusion, LASSO, gene expression, miRNA expression, isform level

## Abstract

Recently, cancer has been characterized as a heterogeneous disease composed of many different subtypes. Early diagnosis of cancer subtypes is an important study of cancer research, which can be of tremendous help to patients after treatment. In this paper, we first extract a novel dataset, which contains gene expression, miRNA expression, and isoform expression of five cancers from The Cancer Genome Atlas (TCGA). Next, to avoid the effect of noise existing in 60, 483 genes, we select a small number of genes by using LASSO that employs gene expression and survival time of patients. Then, we construct one similarity kernel for each expression data by using Chebyshev distance. And also, We used SKF to fused the three similarity matrix composed of gene, Iso, and miRNA, and finally clustered the fused similarity matrix with spectral clustering. In the experimental results, our method has better *P*-value in the Cox model than other methods on 10 cancer data from Jiang Dataset and Novel Dataset. We have drawn different survival curves for different cancers and found that some genes play a key role in cancer. For breast cancer, we find out that HSPA2A, RNASE1, CLIC6, and IFITM1 are highly expressed in some specific groups. For lung cancer, we ensure that C4BPA, SESN3, and IRS1 are highly expressed in some specific groups. The code and all supporting data files are available from https://github.com/guofei-tju/Uncovering-Cancer-Subtypes-via-LASSO.

## 1. Introduction

Numerous studies have shown that cancer is a heterogeneous disease (Wang et al., 2005). Today, doctors can use the special information contained in different cancers for more targeted treatment (Fedele et al., 2014; Fu et al., 2014; Marino et al., 2017). Therefore, it is very meaningful to be able to accurately identify cancer subtypes, including molecular subtyping as well as clinical outcome-based clustering. For breast cancer, four major molecular subtypes include Luminal A, Luminal B, Triple negative/basal-like, and HER2-enriched. However, clustering samples based on therapy response and the aggressiveness level may not overlap with these subtypes. With the development of whole-genome sequencing techniques in recent years, the diagnosis and treatments have gained great development (Wang K. et al., 2014; Haase et al., 2015). We have obtained massive cancer expression from database as The Cancer Genome Atlas (TCGA) (Tomczak et al., 2015). Thus, these expression data have positive influence on the development of the cancer subtype identification tools (Sohn et al., 2017; Guo Y. et al., 2018).

Generally, the machine learning method is now widely used to solve clustering problem for cancer subtypes (Kourou et al., 2015; Li et al., 2016; Mirza et al., 2019). Wang et al. (2018) combined Monte Carlo feature selection (MCFS), random forest (RF), and rough set-based rule learning to identify breast cancer. Li and Ruan (2005) used support vector machine for cancer recognition. Monti et al. (2003) combined resampling consensus clustering. Also, there are many tools based on deep learning method (Wang et al., 2016; Esteva et al., 2017; Miotto et al., 2017). Chen et al. (2019) used RNN to identify some genes that have an impact on cancer. Neighbor Ensemble-based Detection (NED) proposed by Zhou et al. identified lung cancer cells (Zhou et al., 2002). Karabatak and Ince (2009) identified breast cancer through association rules (AR) and neural network (NN). Brunet et al. (2004) proposed non-negative matrix factorization to find cancer subtype.

Furthermore, many predictive models can identify cancer subtypes by using single expression data (Verhaak et al., 2010; Chen et al., 2013; Zhang et al., 2017). Verhaak et al. (2010) employed gene expression to identify four subtypes in glioblastoma multiforme (GBM). Brunet et al. (2004) used gene expression to uncover subtypes on three datasets, including Myelogenous leukemia, Medulloblastomas, and Central Nervous System Tumors. Wong et al. (2012) proposed the Feature Set Reduction method to select more important single nucleotide polymorphism and classify cancer subtypes on three diseases as sarcoma, lymphoma, and leukemia. Zhang et al. (2017) used DNA methylation to find cancer subtypes on breast cancer. Pan et al. (2018) used copy number variants to identify four cancer subtypes on breast cancer. Zhao et al. (2009) used single-stranded DNA (ssDNA) to find cancer subtypes on lung cancer.

However, since cancer is a heterogeneous disease, independent analysis of a single type of data often results in unsatisfactory consequence. Some studies take advantage of various popular multiple kernel learning methods (Ding et al., 2017; Jiang et al., 2018), mainly through the integration of similarity networks among patients from multiple expression data. Wang B. et al. (2014) integrated three expression data, including gene expression, DNA methylation data, and miRNA expression data, to calculate the patient similarity network by using the similarity network fusion (SNF). Ma and Zhang (2017) improved the SNF and proposed the affinity network fusion (ANF) to cluster multiple cancer patients. The unsupervised multiple kernel learning (UMKL) for multiple datasets was proposed by Mariette and Villa-Vialaneix (2017). Jiang et al. (2019) improved the SNF and proposed the similarity kernel fusion (SKF) to combine three expression data including gene expression, isoform data, and miRNA expression data, and first collected five cancer datasets to verify the performance of model. Jiang et al. used the Euclidean distance when constructing the similarity kernels. The dimensionality of DNA and other features is very large. The use of Euclidean distance may have a great impact on the clustering results.

In this paper, we employ LASSO for gene selection and use Chebyshev distance for constructing similarity kernels. The main process of this article is roughly introduced as follows. First, we extract five novel datasets (bladder cancer, blood cancer, brain cancer, ovary cancer, and pancreas cancer) from The Cancer Genome Atlas (TCGA). It's worth noting that each cancer has three expression data, including gene expression, isoform expression, and miRNA expression. Second, we employed LASSO (Tibshiranit, 1996) to identify the high-efficiency gene expression data and fit survival time, in order to achieve the purpose of feature selection. Since the original gene expression data has high dimensions, the high dimensionality of the data has a very negative effect on the clustering results of small sample size. Third, the Chebyshev distance replaces the Euclidean distance to construct the kernel of the patient's similarity, which can further mitigate the impact of the high-dimensional data. Forth, we used similarity kernel fusion (SKF) to fuse three similarity kernels into one synthetical kernel. Finally, we used spectral clustering on the fused kernel to predict the patient's cancer subtype. In the experimental results, we found that our method achieves outstanding *P*-value in the Cox model on five existing datasets and five novel datasets. We also find the survival curve and the heat map preform outstandingly well on each cancer subtype according to our model.

## 2. Materials and Methods

**Figure 1:**
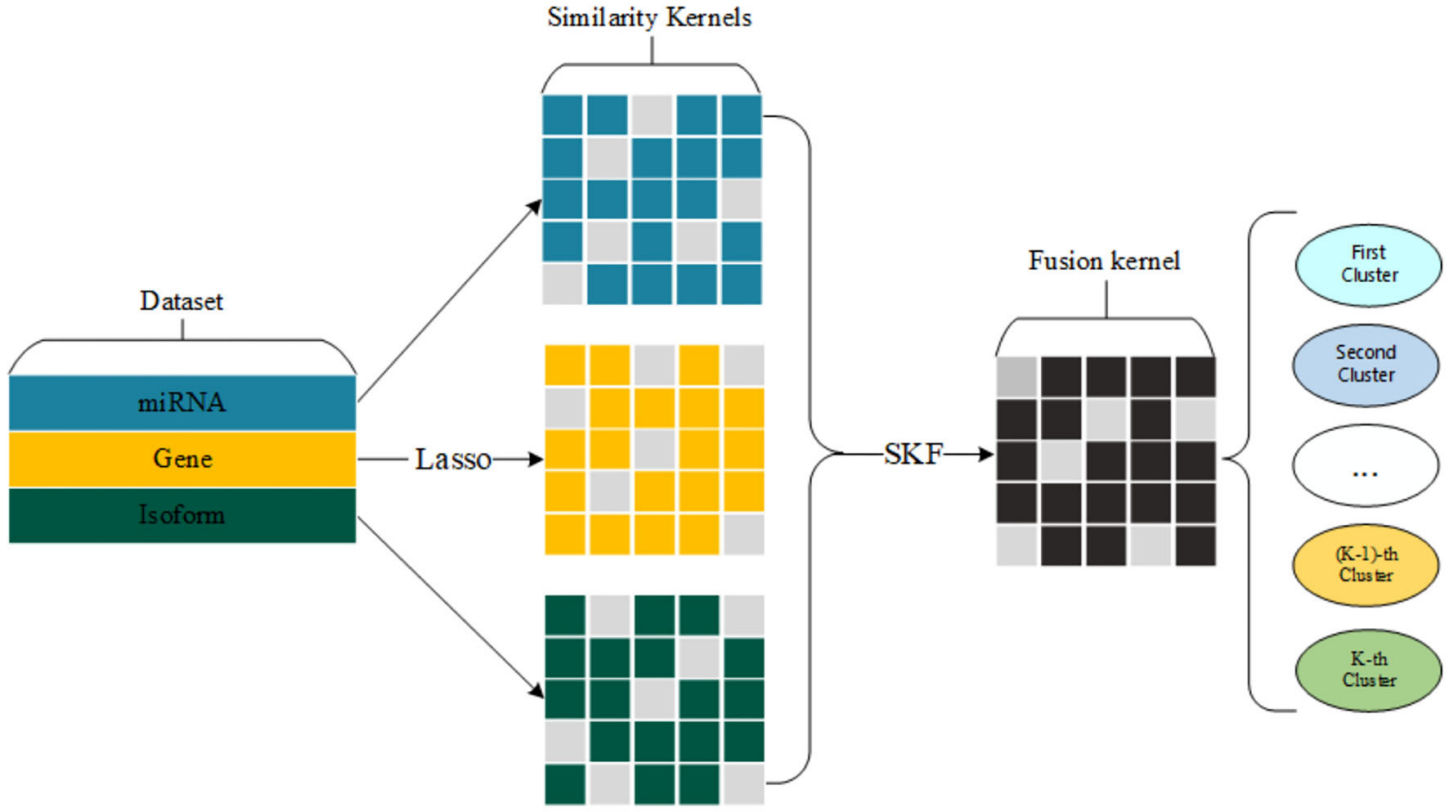
Flow chart of our proposed method for detecting cancer subtypes.

We select a group of significant gene expression to construct three similarity kernels. Also, we fuse three similarity kernels into one kernel for cancer subtype clustering. The whole process of our method is shown in Figure 1.

### 2.1. Novel Dataset

Wang B. et al. (2014) have already extracted five datasets from TCGA, but the number of patients is too small for each dataset. The datasets of Jiang et al. (2019) have alleviated the problem of fewer samples. To better verify the performance of model, we extract five novel data sets, in addition to Jiang's dataset. For each dataset, we select three types of expression data, including gene expression, miRNA expression, and isoform level. The number of expression data is shown in Table 1. We can see that the Jiang's dataset includes stomach cancer, lung cancer, kidney cancer, breast cancer, and colon cancer, Our Novel Dataset add five novel cancer data to Jiang Dataset, which are bladder cancer, blood cancer, brain cancer, ovary cancer, and pancreas cancer.

**Table 1:** Description of Jiang dataset and Novel dataset from TCGA.

	**Disease**	**Patients**	**Gene**	**Isoform**	**miRNA**
	Stomach	1, 071	60, 483	183	1, 881
	Lung	981	60, 483	174	1, 881
Jiang dataset	Kidney	868	60, 483	176	1, 881
	Breast	1, 071	60, 483	183	1, 881
	Colon	426	60, 483	186	1, 881
	Bladder	427	60, 483	211	1, 881
	Blood	165	60, 483	166	1, 881
Novel dataset	Brain	532	60, 483	239	1, 881
	Ovary	374	60, 483	175	1, 881
	Pancreas	177	60, 483	262	1, 881

### 2.2. Gene Selection

The gene expression data have high dimensions in our novel extracted datasets. Due to the curse of dimensionality, high-dimensional data have a great influence on the experimental results. Therefore, We use LASSO to select a part of important genes. We give a formalized description of LASSO, as Equation (1).

(1) $$\begin{array}{l} \min\frac{1}{2}\overset{N}{\underset{n=1}{\sum }}{\left(y\left(X_{n\cdot },\omega \right)-T_{n}\right)}^{2}+\frac{\lambda }{2}\|\omega {\|}_{1} \end{array}$$

We represent patient data as *X* ∈ *R*^*n*×*m*^, where *n* is the number of patients and *m* is the number of expression factors. Patient survival time is defined as *T* ∈ *R*^*n*×1^. We choose the gene expression with the coefficient more than zero as the selected gene features.

### 2.3. Similarity Kernel Construction

We make use of Chebyshev distance (Krivulin, 2011) instead of traditional Euclidean distance to construct the similarity between two patients. The Chebyshev distance is a metric defined on a vector space where the distance between two vectors is the greatest of their differences along any coordinate dimension. The Chebyshev distance between two vectors *p* and *q*, with standard coordinates *p*_*i*_ and *q*_*i*_, is defined as Equation (2):

(2) $$\begin{array}{l} D_{Chebyshev}\left(p,q\right)=\underset{i}{\max}\left(\left|p_{i}-q_{i}\right|\right) \end{array}$$

The expression data are denoted as *E* ∈ *R*^*n*×*m*^, where *n* is the number of patients and *m* is the number of expression factors. The expression data have been centered and scaled to unit variance, as Equation (3):

(3) $$\begin{array}{l} x^{\prime }=\frac{x-\bar{X}}{S} \end{array}$$

where *x* is an element of *E*, *x*′ is corresponding elements of *E* after standardization, $\bar{X}$ is the mean of *E* and *S* is standard deviation of *E*. Here, we denote normalized expression data as *E*′.

Based on the processed expression data *E*′, we construct similarity kernel *K* ∈ *R*^*n*×*n*^ for patients. Here, the similarity between two patients is defined as Equation (4):

(4) $$\begin{array}{l} K_{i,j}=D_{Chebyshev}\left(e_{i},e_{j}\right) \end{array}$$

where *K*_*i,j*_ is the similarity between *i*-th patient and *j*-th patient, *e*_*i*_ and *e*_*j*_ are two vectors of *i*-th row and *i*-th row of *E*′.

Finally, we get three similarity kernels for a special cancer, including similarity kernel $K_{1}\in R^{n\times n}$ by using gene expression, similarity kernel $K_{2}\in R^{n\times n}$ by using miRNA expression, and similarity kernel $K_{3}\in R^{n\times n}$ by using isoform level.

### 2.4. Similarity Kernel Fusion

We construct three similarity kernels for patients in the above section. Then, we use similarity kernel fusion (SKF) to combine these kernels into one kernel K^*^ ∈ ℝ^*n*×*n*^.

First, we construct two kernels *P* ∈ ℝ^*n*×*n*^ and *S* ∈ ℝ^*n*×*n*^ for each similarity kernel, where *P* is a normalized kernel and *S* is a sparse kernel that eliminates weak similarity, as Equations (5) and (6):

(5) $$\begin{array}{l} P\left(i,j\right)=\frac{K_{i,j}}{\sum_{k=1}^{n}K_{k,j}} \end{array}$$

where *P* satisfies $\overset{n}{\underset{k=1}{\sum }}P\left(k,j\right)=1$.

(6) $$S(i,j)=\{\begin{array}{ll} 0 & if\;j\notin N_{i}\\ \frac{K_{i,j}}{\sum_{k\in N_{i}}K_{i,k}} & if\;j\in N_{i} \end{array}$$

where *S* satisfies $\overset{n}{\underset{k=1}{\sum }}S\left(i,j\right)=1$, and *N*_*i*_ is a set of top *k* nearest neighbors of *i*-th patient including itself.

Second, we uncover more information by using multiple iterations (Wang B. et al., 2014), as Equation (7):

(7) $$\begin{array}{l} P_{l}^{t+1}=\alpha \left(S_{l}\times \frac{\sum_{r\neq l}P_{r}^{t}}{2}\times S_{l}^{T}\right)+\left(1-\alpha \right)\left(\frac{\sum_{r\neq l}P_{r}^{0}}{2}\right) \end{array}$$

where $P_{l}^{t}$ (*l* = 1, 2, 3) is the status of *l*-th kernel after *t* iterations, α is a coefficient and satisfies α ∈ [0, 1], and $P_{r}^{0}$ (*r* = 1, 2, 3) represents the initial status of *P*_*r*_.

After *t* + 1 iterations, the overall kernel can be computed as Equation (8):

(8) $$\begin{array}{l} K^{*}=\frac{1}{3}\overset{3}{\underset{l=1}{\sum }}P_{l}^{t+1} \end{array}$$

### 2.5. Mining Subtypes Using Spectral Clustering

Through SKF, we have obtained the fusion kernel containing multi-angle information, and the invention of spectral clustering is to cluster through the kernel. So, We employ spectral clustering on integrated similarity kernel to divide all patients into multiple clusters. In order to ensure that the difference between each pair of classes should be as large as possible, also the similarity within one class should be as large as possible, this problem is a relaxation of the NCut problem (Von Luxburg, 2007). The detailed processes of spectral clustering model is introduced as follows.

First, we calculate the Laplacian matrix *L* based on K^*^. Then, we compute the first *k* generalized eigenvectors {*u*_1_, …, *u*_*k*_} from the generalized eigenproblem *Lu* = λ*Du*, *D* is a diagonal matrix whose diagonal element is the sum of the row elements of K^*^. We define *U* ∈ ℝ^*n*×*k*^ as the matrix containing *k* vectors {*u*_1_, …, *u*_*k*_} as columns, and $y_{i}\in {\mathbb{R}}^{k}$ as the vector corresponding to the *i*-th row of *U*. Finally, we cluster the points {*y*_*i*_}_*i*=1,…,*n*_ in ℝ^*k*^ with the k-means clustering algorithm into clusters {*C*_1_, …*C*_*k*_}.

We define a matrix *Y* ∈ ℝ^*n*×*k*^, *Y*_*j*_ = (*y*_1,*j*_, ..., *y*_*n, j*_) to represent the cluster result (Von Luxburg, 2007), where $y_{i,j}=\frac{1}{vol\left(\sqrt{Cluster_{j}}\right)}$ if patient *p*_*i*_ belongs to *j*-th cluster, otherwise *y*_*i, j*_ = 0. The whole issue can be transformed into solving the optimization problem, as Equation (9):

(9) $$\begin{array}{l} \begin{array}{c} \underset{T\in {\mathbb{R}}^{n\times k}}{\min}Tr\left(T^{\prime }D^{-\frac{1}{2}}LD^{-\frac{1}{2}}T\right)\\ \;s.t.T^{\prime }T=I \end{array} \end{array}$$

where *D* is the degree matrix of K^*^, *L* is the Laplacian matrix of K^*^, $T=D^{-\frac{1}{2}}Y$, $vol\left(A\right)=\underset{i\in A}{\sum }\overset{n}{\underset{j=1}{\sum }}K_{i,j}^{*}$.

Here, our proposed method can be shown in Algorithm 1.

**Algorithm 1:** Algorithm of our proposed method

**Require:** A patient data matrix *X* ∈ *R*^*n*×*m*^, Patient survival time vector *T* ∈ *R*^*n*×1^.
**Ensure:** *Y* ∈ {0, 1}^*n*×*k*^ to represent cluster result, where *Y*(*i, j*) = 1 if patient *p*_*i*_ belong to *j*-th cluster.
1: Feature selection through LASSO, as Equation (1);
2: Normalize X and denote expression data as *E*′;
3: Get the similarity kernels *K*1, *K*2, *K*3 ∈ *R*^*n*×*n*^, as Equation (4);
4: Use SKF algorithm for kernel fusion, as K^*^ ∈ *R*^*n*×*n*^;
5: Minimize Equation (9) to obtain *Y* ∈ {0, 1}^*n*×*k*^.

## 3. Results

In this section, we analyze the performance of our method on the dataset in several ways. First, we introduce an evaluation criteria and a verification method that are used to evaluate the significant performance of cancer subtypes prediction. Second, we analyze the performance of SKF on the Jiang's dataset. Third, we analyze the performance of LASSO on the Jiang's dataset. Fourth, we compare our method with other methods. Fifth, we apply five novel data sets to evaluate our new method. Finally, we plot survival curves and heat maps for some cancers.

### 3.1. Evaluation Criteria

In this paper, we use the *P*-value of Cox regression model and survival curve to evaluate the performance of our method, while the lower *P*-value indicates higher performance significance. Here, we use 0.05 as a standard for evaluating the performance of clustering results. The actual significance of *P*-value is the difference in survival rates among cancer subtypes. In addition, survival curve is the change of survival rate with survival time. We can find from the survival curve that different cancer subtypes have different survival odds. We can focus on cancer subtypes with high mortality.

### 3.2. Performance of SKF

In this section, we compare our approach on the use of SKF with the same model on the use of SNF, UMKL, the average kernel fusion or the direct use of single kernel on the Jiang's dataset. There are two important parameters α and *K* in SKF. We chose *K* = 30 and α = 0.9 through experiments. Because the parameter space is very large, we mainly adjust *K* by fixing α first, and then fix *K* to adjust α to get an a local The optimal value.

#### 3.2.1. Comparing SKF With Single Kernels

On the Jiang's dataset, we separately record the results of using SKF and using a single kernel, as shown in Table 2. We can see that the P value of some diseases is <0.05, despite using the single kernel. However, after using SKF for the kernel fusion, the effects of Lung, Breast, and Colon have been significantly improved. It can be seen that it is necessary to fuse the similarity kernels.

**Table 2:** Comparison between SKF and the single kernel on Jiang Dataset.

**Cancer**	**Gene**	**miRNA**	**Isoform**	**SKF**
Stomach (C = 10)	0.196	0.076	0.327	0.002
Lung (C = 7)	0.005	0.173	0.241	1.586 × 10^−6^
Kidney (C = 10)	0.082	0.642	0.585	0.018
Breast (C = 5)	0.009	0.680	0.322	1.116 × 10^−5^
Colon (C = 11)	0.046	0.050	0.099	1.117 × 10^−6^

#### 3.2.2. Comparing SKF With Different Kernels

In SKF, the choice of kernel is a very important factor. In most cases, we will choose Euclidean distance as the kernel generation formula, but considering that the dimensionality of biological data is generally large, using Euclidean distance will not have a good effect, we choose Chebyshev distance to construct the kernel. Specifically, it can be seen from the Table 3 that choosing the Chebyshev distance has a significant improvement in the results.

**Table 3:** Comparing SKF with different kernels.

**Cancer**	**Euclidean**	**Chebyshev**
Stomach (C = 10)	0.043	0.002
Lung (C = 7)	0.089	1.586 × 10^−6^
Kidney (C = 10)	0.175	0.018
Breast (C = 5)	0.042	1.116 × 10^−5^
Colon (C = 11)	0.130	1.117 × 10^−6^
Bladder (C = 5)	0.147	0.001
Blood (C = 7)	0.805	0.029
Brain (C = 9)	0.040	0.076
Ovary (C = 7)	0.681	0.001
Pancreas (C = 7)	0.243	0.008

#### 3.2.3. Comparing SKF With Other Fusion Models

**Figure 2:**
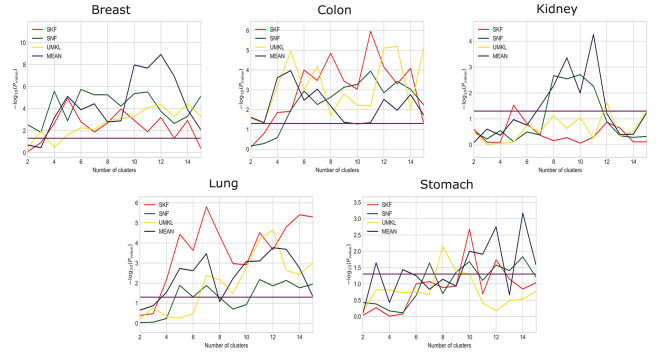
Comparison between SKF and other fusion models.

We compare the results using SKF with the results of SNF, UMKL, and the average kernel fusion, as shown in Figure 2 and *X* axis is the number of clusters and *Y* axis is the value of −log_10_(*P*_*value*_). Red, green, yellow, and purple represent the results of using SKF, SNF, UMKL, and average kernel, respectively. And the horizontal line represents the *p*-value of 0.05. We can see that there is a better performance on Stomach, Lung, and Colon by using SKF. The use of SKF for the kernel fusion on Breast is very similar to that of SNF. It is not as good as SNF on Kidney, but similar to the results of other kernel fusions. Therefore, it can be found that the use of SKF for kernel fusion has an effect on most datasets.

### 3.3. Performance of LASSO

**Figure 3:**
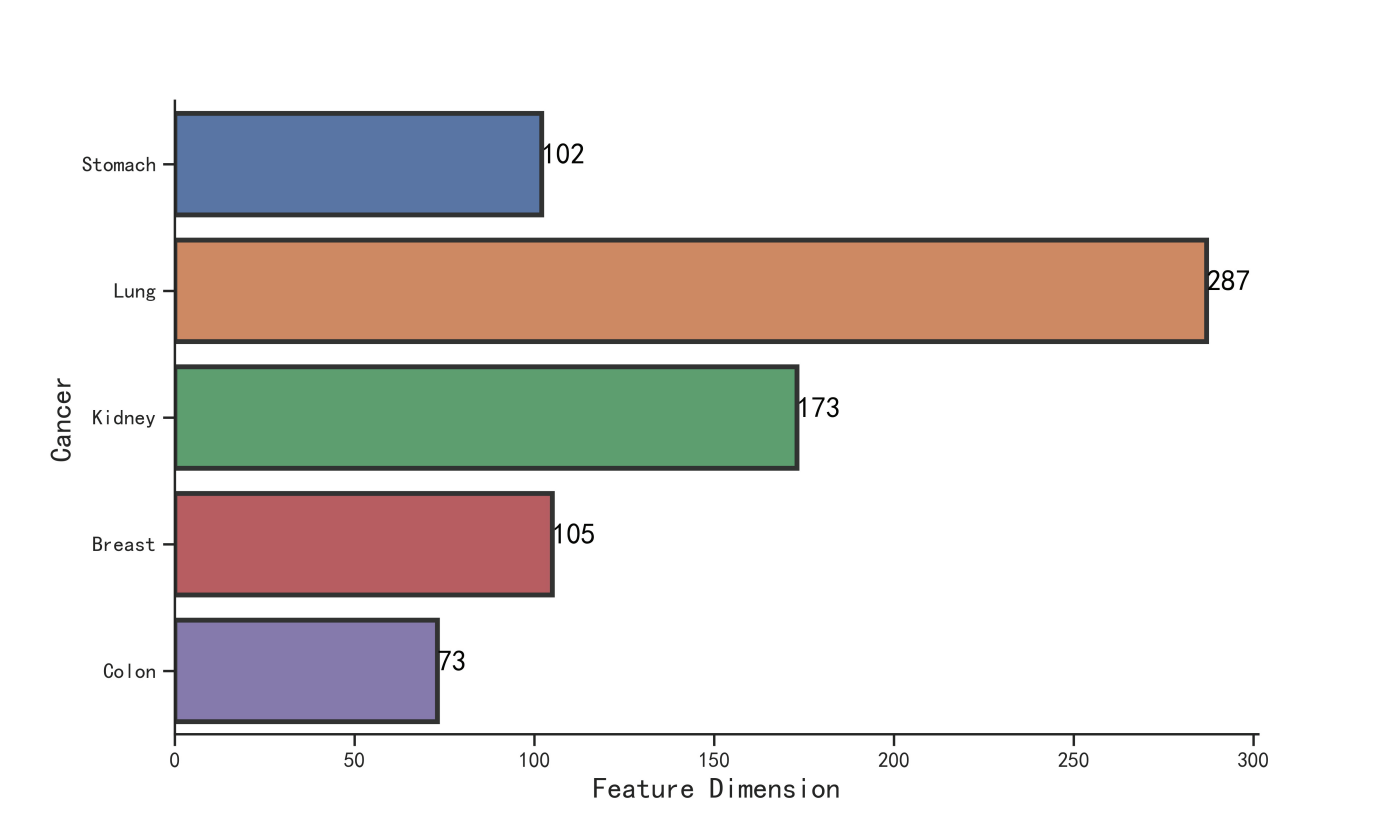
The number of genes selected by LASSO.

**Figure 4:**
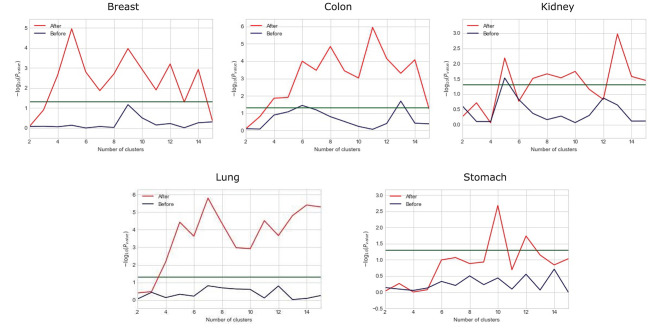
Comparison between the performance of expression data before and after selection by LASSO.

We observe that the original gene expression data has high dimension. Therefore, we use LASSO to identify the high-efficiency gene expression data. In Figure 3, we list the dimensions of gene reduction, and the size of gene is greatly reduced, which is very helpful for later experiments. In Figure 4, we compare the performance of expression data before and after selection by LASSO. The X-axis represents the number of clusters, the Y axis represents −log_10_(*P*_*value*_), the red line represents the data obtained after selection, the blue line represents the data obtained before selection, and the horizontal line represents the *P*-value of 0.05. We can find that the selection of expression data has a certain influence on the *P*-value on Stomach, Kidney, while the *P*-value is greatly improved on Lung, Breast, Colon. Therefore, it can be found that the use of LASSO for selection of expression data has an effect on most datasets.

### 3.4. Comparing With Other Existing Methods

We compare our approach with the method of Jiang et al. (2019), as shown in Table 4. We find that the clustering results of Lung, Kidney, and Colon that using LASSO to select expression data before constructing the kernels and using Chebyshev distance instead of Euclidean distance to construct the kernels, have achieved outstanding performance.

**Table 4:** Performance of different methods on Jiang's dataset.

**Cancer**	**Our method**	**Jiang's method**
Stomach (C = 10)	0.002	8.86 × 10^−14^
Lung (C = 7)	1.586 × 10^−6^	3.81 × 10^−4^
Kidney (C = 10)	0.018	0.120
Breast (C = 5)	1.116 × 10^−5^	6.1 × 10^−6^
Colon (C = 11)	1.117 × 10^−7^	0.025

### 3.5. Performance of Our Method on Novel Dataset

In the above section, our method has outstanding performance on Jiang Dataset. To further evaluate this model, we extract five novel datasets from the TCGA website and apply our method to these novel datasets. The detailed results are shown in Table 5. We can find that our method performs outstandingly well on Brain, and still has good performance on the remaining four diseases.

**Table 5:** Performance of our method on Novel dataset.

**Cancer**	**Gene**	**miRNA**	**Isoform**	**SKF**
Bladder (C = 5)	0.006	0.010	0.378	0.001
Blood (C = 7)	0.011	0.329	0.258	0.029
Brain (C = 9)	1.934 × 10^−7^	0.392	0.585	0.076
Ovary (C = 7)	0.011	0.907	0.167	0.001
Pancreas (C = 7)	0.014	0.507	0.099	0.008

### 3.6. Survival Analysis

**Figure 5:**
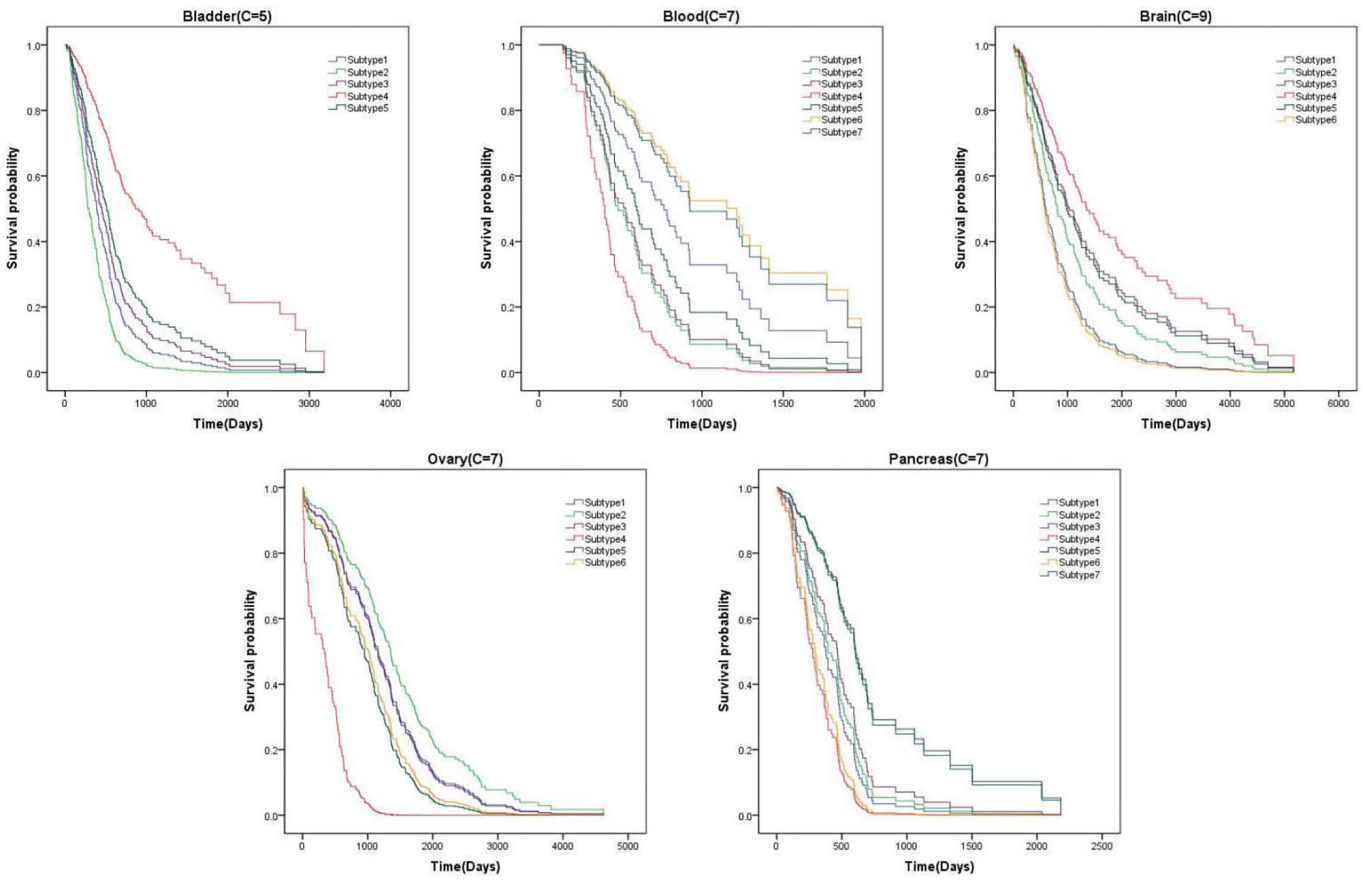
Survival curve on the novel dataset.

From above, we have better measured the performance of clustering results on *P*-value. In this section, We list the survival curves of five cancers on the novel dataset, as shown in Figure 5. We can find that the difference of tendency between each subtype is very obvious on two cancers. It demonstrates that the clustering results have positive guidance for clinical treatment.

### 3.7. Analysis of Essential Genes

**Figure 6:**
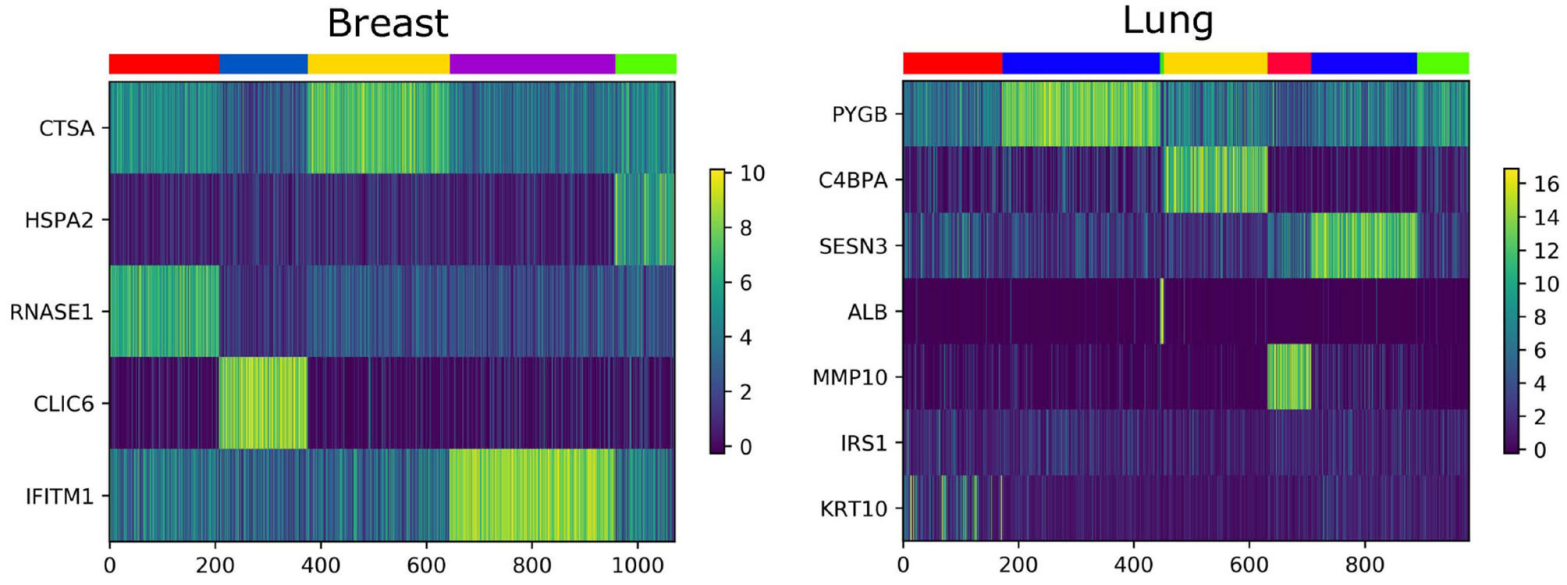
The heat map of essential gene expression data.

We analyze the importance of essential genes on Lung and Breast datasets. The association between clustering results and expression data are shown in Figure 6. The X-axis is the patient, the Y-axis is the gene, and each color of the upper color block represents a category. We find that some essential genes have an effect on the identification of cancer subtypes, most of them can be confirmed by the GEO Profile Database.

**Figure 7:**
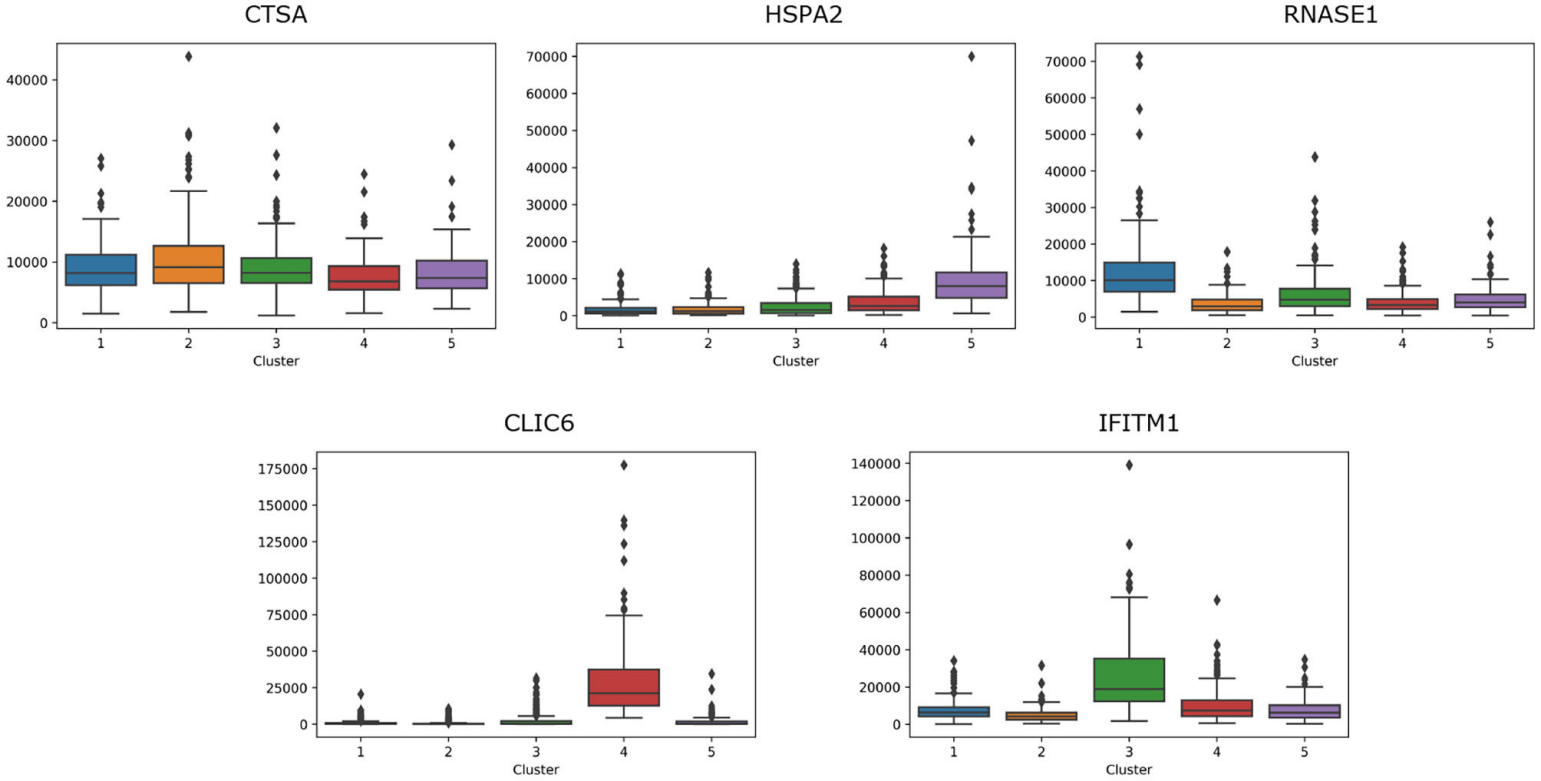
The box plot of essential gene expression data for breast cancer.

For breast cancer, we select five essential genes, such as CTSA, HSPA2, RNASE1, CLIC6, IFITM1. We analyze the box plot of essential gene expression data in five categories, as shown in Figure 7. We find that, HSPA2A is highly expressed in 5-th group, RNASE1 is highly expressed in 1-th group, CLIC6 is highly expressed in 4-th group, and IFITM1 is highly expressed in 3-th group.

**Figure 8:**
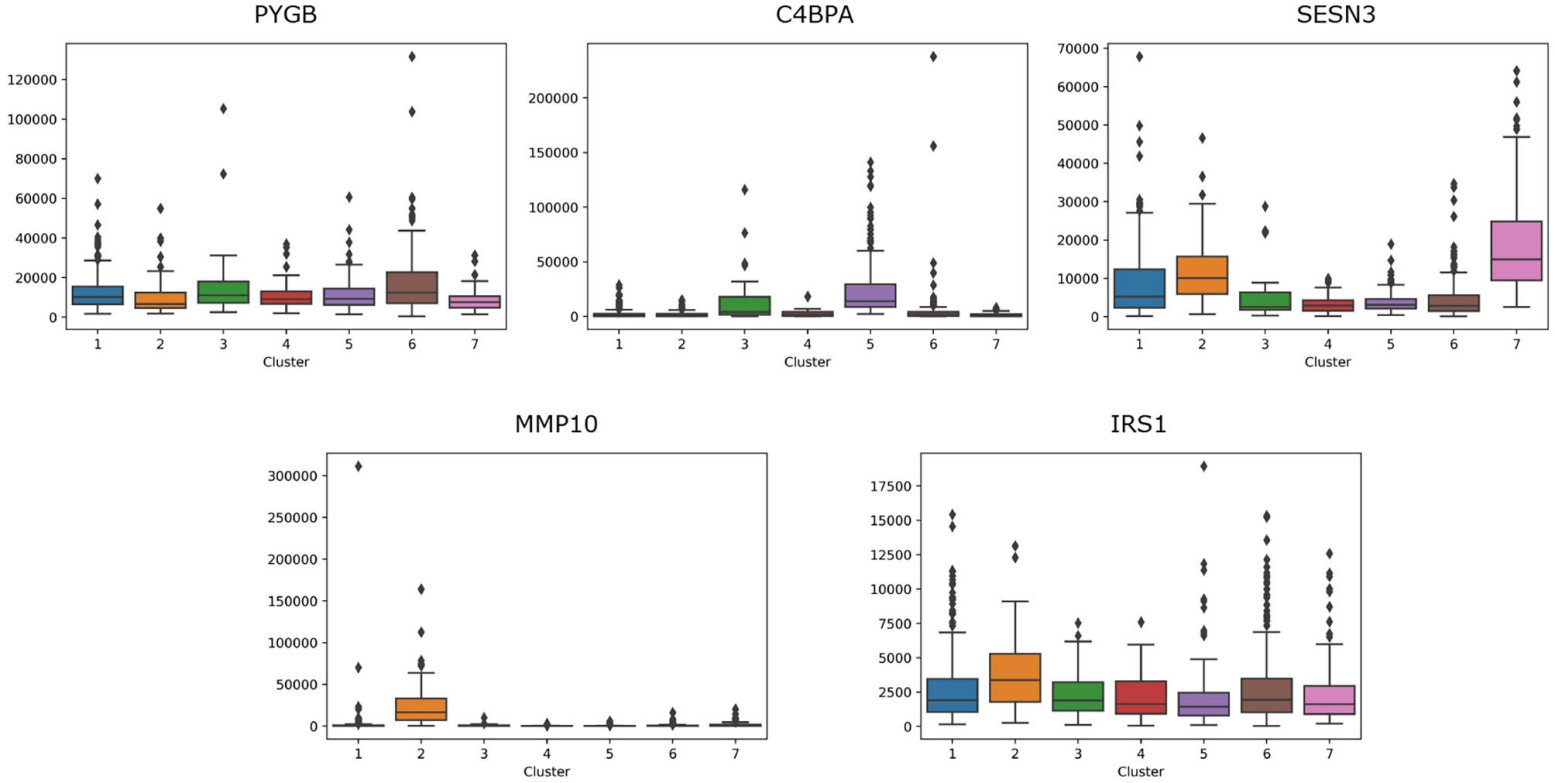
The box plot of essential gene expression data for lung cancer.

For lung cancer, we select five essential genes, such as PYGB, C4BPA, SESN3, MMP10, IRS1. We analyze the box plot of essential gene expression data in seven categories, as shown in Figure 8. We find that, C4BPA is highly expressed in 3-th and 5-th groups, SESN3 is highly expressed in 2-th and 7-th groups, and IRS1 is only highly expressed in 2-th group.

## 4. Conclusion

In this paper, we extract five novel datasets (bladder cancer, blood cancer, brain cancer, ovary cancer, and pancreas cancer) from the TCGA website. We find that our method not only works well on the Jiang's dataset, but also performs well on our newly extracted five datasets. In addition, we obtain some important genes that are related to a special cancer.

In the future, we will try to employ more kinds of expression data to further uncover cancer subtype because cancer is a multi-factors disease (Guo F. et al., 2018). We can also consider other machine learning methods or deep learning methods to uncover cancer subtype rather than spectral clustering (Ding et al., 2019; Shen et al., 2019).

## Data Availability Statement

All datasets presented in this study are included in the article/supplementary material.

## Author Contributions

FG, LJ, and SL conceived and designed the experiments. SL and LJ performed the experiments and analyzed the data. SL, NG, and FG wrote the paper. FG, NG, and JT supervised the experiments and reviewed the manuscript. All authors have participated in study discussion and manuscript preparation. All authors read and approved the final manuscript.

## Conflict of Interest

The authors declare that the research was conducted in the absence of any commercial or financial relationships that could be construed as a potential conflict of interest.
